# Zinc: A small molecule with a big impact on sperm function

**DOI:** 10.1371/journal.pbio.2006204

**Published:** 2018-06-07

**Authors:** Diana S. Chu

**Affiliations:** Department of Biology, San Francisco State University, San Francisco, California, United States of America

## Abstract

Zinc is an essential mineral, but our understanding of its uses in the body is limited. Capitalizing on approaches available in the model system *Caenorhabditis elegans*, Zhao and colleagues show that zinc transduces a signal that induces sperm to become motile. This is an enigmatic process because sperm in all sexually-reproducing animals are transcriptionally inactive. Zinc levels inside sperm are regulated by an evolutionarily conserved zinc transporter called Zrt- and Irt-like Protein Transporter 7.1 (ZIPT-7.1). This zinc transporter localizes to intracellular organelles, suggesting that it primarily controls zinc levels by releasing zinc into the cytoplasm from internal stores rather than importing it from the external environment. The zinc released within cells acts as a messenger in a signaling pathway to promote mobility acquisition. These studies reveal an important role for zinc as an intracellular second messenger that generates physiological changes vital for sperm motility and fertility.

## Introduction

Many of the mysteries of human reproduction are buried deep within the organs of our bodies. Such is the case with how the highly specialized sperm cell forms within the testes. Each cell becomes streamlined and motile to efficiently deliver its tightly wrapped DNA package to an awaiting oocyte. Sperm formation and function are critical to fertility—defects in sperm quantity, quality, and motility account for up to 50% of infertility cases and may affect up to 7% of all men [1]. However, our basic understanding of sperm development and function is lacking, leading to a dearth of knowledge about how problems arise that cause infertility.

**Fig 1:**
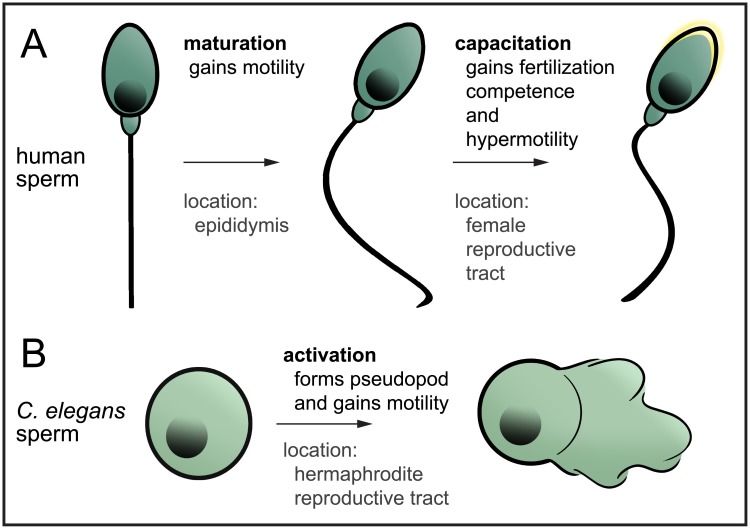
Schematic representations of stages of sperm motility activation. (A) In humans, sperm are formed during spermatogenesis in the seminiferous tubules but are not motile nor competent to fertilize. During transit and storage in the epididymis, they undergo maturation to gain the ability to move. Upon delivery into the female reproductive tract, sperm become capable of fertilization through a process called capacitation, which alters the sperm head membrane to allow for membrane fusion and causes the sperm to become hypermotile. (B) In *C*. *elegans*, sperm are formed during spermatogenesis in both hermaphrodites and males. When males mate to hermaphrodites or when hermaphrodites switch to oocyte formation, sperm become activated. This activation causes the formation of the pseudopod that allows the sperm to crawl.

The formation of sperm is carefully staged in different regions of the testes [2]. Human sperm are first formed in seminiferous tubules, where DNA is partitioned and then tightly compacted; unnecessary cellular components are eliminated; and cells differentiate. These changes form a compact and protected package with a long flagellum (Fig 1A). However, these sperm cannot move or fertilize. They acquire these abilities through signals they receive externally that must be transmitted through the cell without transcription, which is shut down due to tight compaction of sperm DNA [3]. Motility is enabled while sperm “mature” by traversing through the epididymis, a network of coiled tubules that, when stretched out, measure several feet in length [4] (Fig 1A). Within these tubules, sperm are bathed in fluids that contain maturation signals that prepare them for delivery to the female [5]. Once delivered, they further activate through a process called capacitation by exposing receptors important for fertility and becoming hypermotile [6]. Unfortunately, the inaccessibility of reproductive tissues has hindered our understanding of the molecular nature of components that generate or convey signals that contribute to these transformations.

The need for sperm to mature or activate during a period of transcriptional inactivity is common amongst sexually-reproducing animals, even simple ones with very different-looking sperm. These less complex organisms have long been ideal for the study of male fertility, as they allow the use of methods not easily conducted in humans. One such organism is the small nematode *Caenorhabditis elegans* [7]. *C*. *elegans* is transparent; thus, researchers can easily view spermatogenesis in males or hermaphrodites and fertilization of oocytes within hermaphrodites [8,9]. *C*. *elegans* amoeboid sperm, unlike the flagellar-propelled sperm of mammals, crawl using an appendage called a pseudopod [10]. However, just like mammalian sperm, *C*. *elegans* sperm must also receive signals to cue the formation of their motility apparatus, the pseudopod, which becomes active as soon as it is formed (Fig 1B).

In the 1970s and 1980s, *C*. *elegans* researchers conducted mass genetic screens that identified dozens of genes that were defective in spermatogenesis (*spe*) or fertilization (*fer*) when mutated [11,12]. One example is the *spe-8* gene that encodes a protein tyrosine kinase, whose family members relay cellular information via phosphorylation [13,14]. Several other SPE proteins also function with SPE-8, forming the SPE-8 signaling pathway that, though active in both sexes, is essential in hermaphrodites for pseudopod formation and motility [15–18]. More recent genetic screening also identified a distinct male sperm activation pathway triggered by a protease delivered by males along with sperm [19]. However, significant gaps remain in our understanding of the SPE-8 hermaphrodite signaling pathway. This includes how this pathway is activated and how the signal is propagated within cells to make sperm motile. Researchers have been combing through mutants identified by fertility screens to find these missing links but have yet to put all the pieces together to define the entire pathway.

A surprising candidate pathway member—zinc—was found by an in vitro method of isolating immature *C*. *elegans* sperm and exposing them to compounds [20,21]. High levels of extracellular zinc or the activation of the SPE-8 pathway caused intracellular zinc levels to redistribute. These studies suggested that zinc may initiate the SPE-8 signaling cascade or function within the cascade to activate sperm. However, the molecular details of exactly how zinc is acting in a signaling pathway—as the initiation signal or as a signal propagator—were unclear.

The stories converged when three research groups realized that they were working on the same protein—a zinc transporter [22]. The Kornfeld and Ellis labs were looking for proteins that resemble the highly evolutionarily conserved ZIP proteins, which are named after the yeast Zrt- and Irt-like protein zinc transporters [23]. They found that deletion of one of these homologs, *zipt-7*.*1*, caused sterility. Meanwhile, the Singson lab was looking for a sperm activation signal by screening for fertility mutants. They found a mutant with a lesion in the same gene discovered in one of the original fertility screens known as *hc130*. Sequencing *hc130* animals confirmed that they harbor a mutation in the *zipt-7*.*1* gene.

The labs worked together to determine how zinc and the ZIPT-7.1 zinc transporter fit into a signaling pathway required for fertility: Is zinc an external signal for activation or an internal messenger of the signal? One clue about ZIPT-7.1 function is that this transmembrane protein is localized within early developing sperm cells, indicating a possible function on internal membranes. Further, when *C*. *elegans zipt-7*.*1* is expressed in mammalian cells, it also localizes to regions that overlap with intracellular organelles. The authors show that ZIPT-7.1 functions in regulating zinc levels in cells: *C*. *elegans* mutants without *zipt-7*.*1* have lower levels of internal zinc, which is stored in internal organelles, and mammalian cells expressing the *C*. *elegans zipt-7*.*1* show an increased rate of zinc uptake in the presence of externally added labeled zinc.

To further show that ZIPT-7.1 functions inside of cells, the authors determined where ZIPT-7.1 is functioning within the SPE-8 pathway. They found that ZIPT-7.1 functions downstream of a pathway member—SPE-6, known to function within the cell [15]—and interacts with another member called SPE-4, which also localizes to internal membranes [24]. This places ZIPT-7.1 at the end of the SPE-8 pathway to regulate the release of zinc into the cytoplasm from internal stores to propagate the activation signal. The authors could not rule out that zinc also plays some role in extracellular signaling but posit that high levels of extracellular zinc may mimic intracellular release, bypassing much of the SPE-8 pathway. However, with zinc and ZIPT-7.1 clearly having intracellular roles, it is likely that the activating signal of the SPE-8 pathway is still yet to be elucidated.

**Fig 2:**
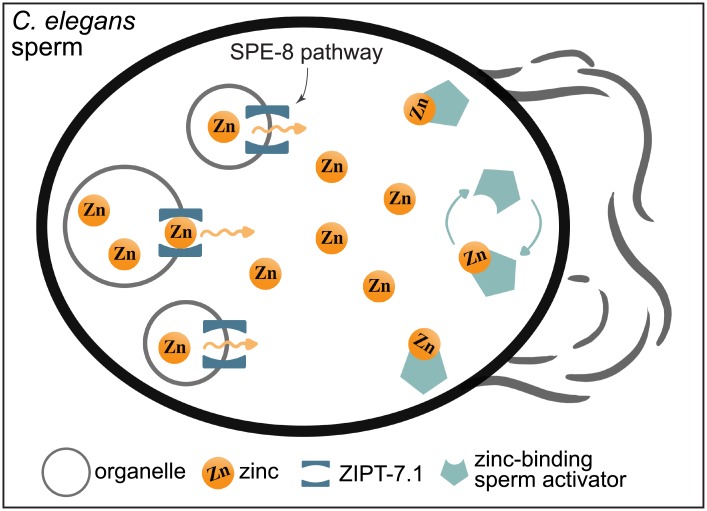
Model for how zinc functions as a second messenger during *C*. *elegans* sperm activation. Upon activation by the SPE-8 signaling pathway, zinc is released into the cytoplasm from intracellular storage organelles via ZIPT-7.1. High levels of cytoplasmic zinc activate yet-to-be-identified zinc-binding proteins that trigger the physiological changes to develop motility structures. SPE-8, spermatogenesis defective; ZIPT-7.1, Zrt- and Irt-like Protein Transporter 7.1.

The model for this pathway places the ZIPT-7.1 protein on the membranes of internal organelles that store zinc in inactive sperm. When sperm receive the still-mysterious signal that activates the SPE-8 pathway, ZIPT-7.1 becomes active and releases the zinc from intracellular organelles into the cytosol. High levels of cytoplasmic zinc presumably activate zinc-regulated proteins that develop motility structures in the absence of transcription (Fig 2). This places zinc as an important “second messenger” that relays the activation signal to intracellular proteins that modulate motility acquisition.

The findings of this paper are novel because they show that zinc has a distinct role as a second messenger in a defined biological signaling pathway vital for fertility. Zinc, an essential mineral, has well-established roles in stabilizing the structure and enzymatic activity of specific classes of zinc-binding proteins, like transcription factors. However, the lack of transcription at this stage of sperm development makes it unlikely that zinc acts to promote transcription. Instead, this study reveals how zinc levels are controlled and read by the cell. (1) Zinc levels inside the cell are highly regulated because the levels are read to change the activity of cellular proteins. (2) Zinc transporters are critical to regulating levels of zinc inside the cell, indicating they can regulate the release of zinc from internal stores into the cytoplasm, not just the import of zinc from the external environment. (3) Male fertility depends upon second messengers like zinc to induce physiological changes in sperm during a critical period where transcription is not active.

This study indicates that roles for zinc and zinc transporters in signaling may be important to investigate in human sperm development and function. Though calcium has long been known to function as a signaling component important for fertility [25], other reports are surfacing of zinc playing signaling roles in various contexts. For example, a release of zinc from oocytes into the extracellular space, referred to as a zinc spark, has been shown to occur upon fertilization as eggs activate [26]. Intracellular zinc can also regulate calcium release in cardiac cells [27]. For male fertility, zinc levels are high in the testes, and zinc transporters are expressed in different regions of the epididymis [28,29]. Further, zinc deficiency is correlated with decreased male fertility [30,31]. Because zinc is so abundant in the labyrinth of testicular tubules, roles for zinc in male fertility must still be untangled. However, these studies demonstrate that investigating roles for intracellular zinc mediating developmental transformations will be an important avenue to explore for numerous processes across a broad range of species.

## Abbreviations

SPE: spermatogenesis defective
ZIPT-7.1: Zrt- and Irt-like Protein Transporter 7.1
